# The Conservation Physiology of Bryophytes

**DOI:** 10.3390/plants11101282

**Published:** 2022-05-10

**Authors:** Marko S. Sabovljević, Marija V. Ćosić, Bojana Z. Jadranin, Jovana P. Pantović, Zlatko S. Giba, Milorad M. Vujičić, Aneta D. Sabovljević

**Affiliations:** 1 Institute of Botany and Botanical Garden, Faculty of Biology, University of Belgrade, Takovska 43, 11000 Belgrade, Serbia; marijac@bio.bg.ac.rs (M.V.Ć.); b3029_2019@stud.bio.bg.ac.rs (B.Z.J.); jpantovic@bio.bg.ac.rs (J.P.P.); zgiba@bio.bg.ac.rs (Z.S.G.); milorad@bio.bg.ac.rs (M.M.V.); aneta@bio.bg.ac.rs (A.D.S.); 2 Department of Botany, Institute of Biology and Ecology, Faculty of Science, Pavol Jozef Šafárik University in Košice, Mánesova 23, 040 01 Košice, Slovakia

**Keywords:** mosses, liverworts, threaten species, functional traits, protection

## Abstract

An introduction to the conservation physiology of bryophytes is given. The insights into the problems, solutions and examples of the physiological approach to conservation within bryophyte representatives are discussed. The significance of experimental treatments of bryophytes is highlighted. The documentation of bryophyte functional traits and eco-physiological mechanisms in the conservation background for protection purposes is highlighted by the selected examples. The introduction of bryophytes into a new scientific field is resumed and some insights from specific case studies are presented.

## 1. Introduction

Conservation physiology is a rather new scientific discipline emerging over the last several decades with the aim of solving the conservation problems of different biological entities. This is an integrative approach applying physiological concepts and tools to gain new knowledge about the features of those targeted biological entities which are the subject of conservation. Once conservation is needed, we usually know very little about the entity (organisms, populations or ecosystems), and lack crucial data pertaining to the functional characteristics of the biological entity and its responses to environmental stressor or changes. Thus, in order to address conservation problems, data on the functional responses and, thus, survival strategies in different environmental backgrounds are urgently needed, and conservation physiology provides the opportunity to gain such knowledge directly and quickly through an experimental approach, since many biological entities are in need of urgent conservation and have no time to wait for data accumulation. Additionally, these data are necessary to develop good conservation policy, ecosystem restoration, population rebuilding and self-sustainability or simply to generate the support tools for decision-makers.

Recognition of the significance of physiology for conservation has increased considerably in recent decades, however, mostly in terms of big animal conservation, mainly mammals, and lately also for some plants. However, angiosperms are underrepresented in strict conservation physiological studies and this appears to be a general trend in conservation science [1]. Thus, this approach is missing among many other threatened biological entities (including bryophytes) and further development is both essential and urgent. Plants in general are primary producers, and their importance is indispensable for all other organisms. Van Kleunen science [1] reported 47% of all globally threatened organisms to be among the higher (vascular) plants. Bearing in mind that many plant scientists deal with the physiological responses of model organisms or crop plants, very little information on environmental response mechanisms can be found for wild and threatened plants. The situation with bryophytes (higher but non vascular plants) is even worse.

Thus, information on the mechanisms involved in how biological entities function is urgently needed for threatened taxa, and these include a wide range of areas, such as structure, resource acquisition, metabolic pathways, energy fluxes, regulation and homeostasis, adaption and the ability to tolerate environmental changes.

Some provenance trials on conservation physiology have been applied during ex situ conservation studies, as ex situ and conservation physiology can but do not overlap to a huge extent and are a compatible field in conservation science. Some of the published papers clearly fit into this view as independent research areas whose results support studied entity conservation (e.g., for bryophytes: [2,3,4,5,6,7]), although the history of ex situ conservation studies is relatively short [8]. Ex situ studies often focus on habitat variation limits and suitability for the target biological entity of high conservation interest (e.g., [9]). 

The position of conservation physiology within conservation biology can, thus, be considered as an independent subdiscipline, but one which partly overlaps and intermingles with conservation ecology and genetics. Such a discipline is urgently needed for better conservation planning and management. 

Bryophytes, a group of photosynthetic organisms which were among the first to colonize the terrestrial environment, are rather neglected in conservation initiatives world-wide as being of less economic importance. However, this group of 18,000–25,000 recent species has high significance in ecosystem functioning and also biotechnological potential, thus deserving greater attention in conservation initiatives. 

Although they have many similarities with vascular plants, there are more dissimilarities and peculiarities of this heterogeneous group and the knowledge gained about vascular plant species often leads to misinterpretation or even incorrect conclusions.

The evolutionary distance as seen in years among certain species within the bryophyte group is bigger than that among remote entities of vascular plants. Thus, it can be inferred that even the extrapolation of knowledge among some bryological entities can be misleading. Considering that conservation physiology can include studies on a wide range of scales, from chemical contents or biomolecules through cells and special organs to whole organism and population biology, even more caution should be exercised when inferring measures for tentative species at survival risk.

Carey [10] stated that in addition to environmental changes in plant conservation physiology, special attention should also be paid to pathogen emergence, and we have almost no idea about bryophyte pathogens, thus indicating this need in the emerging field of bryophyte pathogen biology.

In general, bryophytes have a larger area or occupancy, i.e., range compared to vascular plants, but being haploid organisms and highly specialized, they are attached to microhabitats and react easily to minor environmental changes. That is why they have a high indicative potential for environmental changes. On the other hand, this can provide the opportunity to study the variation of physiological traits over large spatial and temporal scales. Chown et al. [11] defined this approach as macrophysiology, which is important in mitigating population decline and plasticity to cope with environmental changes. This is crucial when choosing individuals for studies as well as for captivity and ex situ conservation programs.

In organisms other than bryophytes, accumulated physiological knowledge has allowed for the generation of management models of target entity. Moreover, it has become possible to predict the response to changes, to test and develop conservation strategies, i.e., to reach the goal of desirable conservation outcomes [1].

The approach to various threatened taxa cannot be the same bearing in mind that some suffer from habitat changes and degradation and others from rather low fitness. Although the study of such taxa can be the same, the conservation programs may differ to achieve the same goal.

It should be stated that conservation physiological studies should not be solely directed at environmental changes, but also antropogenically induced impacts and the responses of bryophytes to them. It should be highlighted that conservation physiology and its approach do not include only threatened taxa and abiotic stressors, but also the study of the functions and mechanisms of taxa which may threaten natural ecosystems and native species, such as alien or invasive species.

The conservation physiology of bryophytes faces numerous constraints. There are not very many bryophyte scientists and few of them deal with the experimental approach. The plant material is rather hard to recognize and find in relevant developmental phases, and the known habitat can be very distant and not easily accessible, especially for priority and/or targeted species. In vitro and ex situ collections are rather rare and hard to establish, and the asexual reproduction of haplotypes can be problematic when considering the genetic loss or structure of the population for conservation. Nevertheless, there are also advantage such as the small size of bryophytes compared to other plants, demanding less laboratory space and lower costs.

The developmental traits of bryophytes are not the same for some growth phases and some developmental stages will be absent even when the optimal conditions for vegetative growth are achieved. Thus, studies of the various physiological responses of different growth phases (e.g., sex organ development) are needed as well as the variability of functional traits, referred to as physiological diversity of biological entities. Physiological diversity seems to be extremely important for establishing self-sustainable populations and reintroduction programs in general. Thus, the discovery of the mechanistic basis should lead to functional patterns, which can be achieved through the experimental approach in conservation physiology. The overlapping of data from realized niches usually studied in distributional investigation should be strengthened by the data gathered in fundamental niche studies, hence improving the prediction of survival in a changing environment. Suffering the fitness consequences, but still with some survival rate, is due to a poor understanding of environmental thresholds and organisms’ tolerance of extremes, synergisms and antagonisms of both biotic and abiotic factors in areas of survival but of lower habitat quality.

Physiological tools are useful in defining the areas of the highest functional, and not only structural basis, and so take priority in spatial protection since protecting all habitats is impossible and unrealistic even for the most threatened and/or the rarest species.

Future environmental change scenarios include multiple stressors and physiological approaches and experimental tests enable valid prediction and timely protection. Additionally, physiological tools allow the study of potential pollutants and bryophyte responses to them, thus identifying the potential thresholds for those emerging or long-term present in potential or native habitats.

Although ex situ conservation efforts are often a last resort for the conservation of highly endangered species, they remain an important safeguarding tool. However, many problems and disadvantages emerge when dealing with bryophytes ex situ: limited material availability, a lack of information on biology and ecology, unknown, undeveloped or underdeveloped biotechnological procedures for propagation or appropriate morphological development and desired developmental stage achievement. Additional problems include germplasm formation and maintenance, spore production and storage, unknown spore biology (e.g., dormancy) and nutritional or species-specific requirements. Lack of knowledge about natural enemies, and interaction with other organisms or chemical constitutions make in vitro tests and collection unavoidable when dealing with bryophyte conservation physiology and compiling protection action plans, which include minimized stress and maximized survival.

## 2. Some Examples of Incidental and Intentional Bryophyte Conservation Physiological Approaches in Europe

The conservation of bryophytes is usually conducted through an assessment of species and their populations in their areas of occupancy, resulting in red data books or red lists e.g., [12] or regional or national legislation. There are fewer examples which include the organized monitoring of local populations of target species, and the number where the experimental approach is applied, either via field or laboratory experiments, is even lower. However, bryophyte scientists and enthusiasts are aware of the significance of the experimental approach and experimental evidence when dealing with tentative or sensitive species remaining in small numbers in nature. Thus, in many red-list, red-book and conservation programs, the urgent need for such data on the target species is highlighted. There have been very few experimental approach examples during ex situ conservation, and these tend to identify new problems instead of offering explanations and solutions.

**Figure 1:**
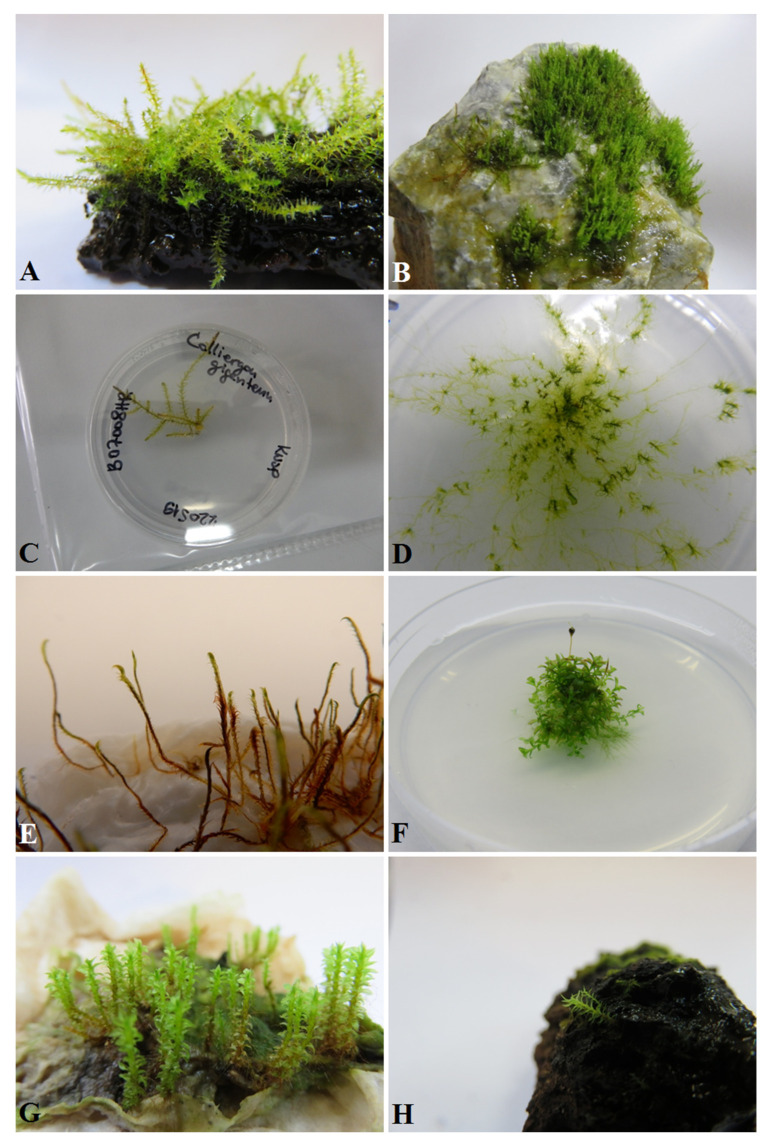
Some examples of moss species applied in conservation physiology programs: (**A**). *Anacamptodon splachnoides* (origin from Hungary) from in vitro culture anchored to the natural wooden substrate; (**B**). *Anomodon rostratus* (origin from Serbia) from in vitro culture anchored to the limestone rocks by the application of egg white; (**C**). *Calliergon giganteum* (origin from Croatia) in vitro propagation; (**D**). *Dicranum viride* (origin from Hungary) in vitro propagation; (**E**). *Hamatocaulis vernicosus* (origin from Romania) xenic condition propagation and acclimation; (**F**). *Physcomitrium eurystomum* (origin from Croatia) propagation in in vitro controlled conditions and sporophyte development with viable spores; (**G**). *Tayloria froelichiana* (origin from Slovakia), xenic propagation and acclimation; (**H**). *Zygodon forsterii* (origin from Hungary) anchoring to wooden substrate and gametophore induction in xenic conditions.

Experimental investigations on bryophytes are avoided because of the problems involved in in treatments and bryophyte material collection and propagation. The studies of in vitro cultures are conducted, but on a small number of species which serve as models (e.g., *Physcomitrium patens* (Hedw.) Mitt., *Marchantia polymorpha* L. or *Ceratodon purpureus* (Hedw.) Brid.). A similar approach is rarely seen for other species (Figure 1). The reasons for this are manifold: from problems in establishing axenic cultures and growth control, to the slow growth of bryophytes, to difficulties in finding a sufficient quantity of clean (free of other cohabitants) target species and their identification. The problems of in vitro culturing bryophytes as well as some solutions and procedures are addressed in some studies e.g., [13,14,15,16] and the references therein. Additionally, more details on procedures and strategies can be found in Sabovljević et al. [14].

Studies which directly address bryophyte conservation problems are rare, but significant contributions can be found. In this chapter, we provide an overview of some of the most interesting instances.

The development of bryophytes is often directed by inner and outer signals, although very little information is available on the developmental physiology of bryophytes. Most extrapolations are based on the knowledge accumulated about vascular plants and a few are derived from the study of moss model species *Physcomitrium patens* (Hedw.) Bruch & Schimp. A review of plant growth regulators in bryophytes can be found in Sabovljević et al. [17]. Very little is known about the differences among species of bryophytes groups and some tests in various species seem to show rather different developmental patterns and functions. This is to be expected considering that the phylogenetical distance between different groups and species can be very great. The absence of vascular systems is common, and the effects of exogenously applied plant growth regulators may serve as developmental triggers [13,18,19]. They can also act as elicitators or blockers depending on the concentrations applied and on the synergistic/antagonistic effects with other tested factors (i.e., chemicals, light conditions or temperature). These findings can be a good starting point in testing the biological features of rare species. This means firstly applying such tests on more common species (counterparts), further developing the tests and then using them on the target species. The selection of counterparts should be in accordance with ecological, physiological or morpho-anatomical characteristics, which should be similar or like those of the target species based on the information available for the target species (elaborated in [14]). Such tests were conducted in *Atrichum undulatum* (Hedw.) P. Beauv. [20], *Bryum argenteum* Hedw. [18], *Dicranum scoparium* Hedw. [21], *Hypnum cupressiforme* Hedw. [22] and *Thamnobryum alopecurum* Nieuwland ex Gangulee [23]. These were later applied to target rare or threatened species, such as *Bruchia vogesiaca* Schwaegr. [24], *Calliergon giganteum* (Schimp.) Kindb. [25], (Figure 1C), *Entosthodon hungaricus* (Boros) Loeske [26] and *Molendoa hornschuchiana* (Hook.) Limpr. [27], to achieve good development and propagation in laboratory conditions prior to testing in outdoor environments. 

The selection of media types for both axenic and xenic growth is not an easy task, so in order to achieve adequate bryophyte development, one should start with the minimal ones [28]. Media type contents are important for various developmental phases, such as spore germination, brachycyte or tmema cell development and promoting gametophore or protonemal growth. Some specific habitat types, such as salty grasslands or gypsiferous outcrops known to bear rare bryophyte species, are in fact selective ecological combination for such species. Namely, species tied to such habitats (e.g., salty grasslands) are shown to be facultative bryo-halophytes, since they can thrive rather well outside such habitats in the absence of other competitive species. For example, the rare *E. hungaricus*, found only in salty soils, can grow rather well in different substrates without salts in in vitro conditions, but also on different tested non-salty soil types, but only in the absence of other species, which without salt easily overtake the spaces and overgrow *E. hungaricus* (e.g., *Barbula unguiculata* Hedw. can quickly overgrow *E. hungaricus* in the absence of salt in the substrate) if the soils are not cleaned of viable spores and diaspores of other moss species (by means of sterilization processes, for example) [29]. On the other hand, while some bryophyte species are known to settle on strictly gypsiferous rock outcrops (areas rich in gypsum) and are ecologically known as bryo-gypsophytes, tests carried out on other species show them to grow rather well on media containing gypsum, suggesting some other limiting factors. Tests carried out with two different non bryo-gypsophytes show them to be indifferent to gypsum concentration, and that some other factor is the limiting one to settle and live only in gypsum habitats, e.g., water supply [30]. The rare and threatened Bern Convention moss *B. vogesiaca* develops large circles of protonemata when growing on BCD media with the addition of IBA, while this does not occur on half strength MS media type enriched with sucrose even if IBA is applied [24]. The tests conducted on the rare and threatened *Goniomitrium seroi* Casas, which thrive well in in vitro axenic laboratory condition, with the aim of inducing tuber formation present in native plants (an important spreading structure), have remained unsuccessful up to date [31]. Similarly, any treatment applied in laboratory conditions to the protonemal filaments of the rare and Bern Convention species *Buxbaumia viridis* (Moug. ex Lam. Et DC.) Brid. ex Moug. et Nestl. lacked any new structural development other than secondary protonemal spread (Sabovljević et al., unpubl. results). Some species developed well, but released too many phenolic compounds into the media, suggesting suboptimal growth conditions and often demanding transfer to new media (e.g., *Entosthodon muhlenbergii* Fife, [32]).

In general, stress caused by abiotic factors in bryophytes can be regarded as responsible for population decreases in nature. The studies document conditional effects, and although many can be found for model moss *P. patens* e.g., [33,34], far fewer experimental approaches can be found for other bryophyte species. Thus, the findings become surprising when we start dealing with rare and threatened species. Bryo-halophyte *E. hungaricus* can thrive very well in non-salty environments, but in such habitats, it is less competitive compared to other mosses [29]. Additionally, different strategies among bryo-halophytes from the same habitats, namely *E. hungaricus* and *Hennediella heimii* (Hedw.) R. H. Zander, can be seen in salt-stress survival [35,36,37,38], differing between species in chemical content quantity and quality when growing in the same controlled conditions, and applying them in salt-stress tolerance and resistance. The state of knowledge on salt stress in bryophytes can be found in more detail in Ćosić et al. [39], and bryophytes have already been reported to have different responses to salt stress to those present in poikilohydric ferns, for example [40]. The addition of nitrogen and phosphorus to wet paper was enough to get well-developed rare and endangered *Tayloria splachnoides* (Schwägr.) Hook. and *T. froelichiana* (Hedw.) Mitt. ex Broth. to grow xenically (Sabovljević et al., unpubl. results, Figure 1G). These are species of very narrow habitat type, such as animal excrement and remains.

The lack of knowledge goes beyond the salt influence when dealing with brackish water liverworts *Riella helicophylla* (Bory et Mont.) Mont. Sabovljević et al. [41] developed bi-phase axenic in vitro systems, a solid one for spore germination and a liquid form for gametophore development. When combined, the solid form containing minerals and essential salts, and the water cover was not enough to achieve vigorous development. The imitation of brackish water conditions, i.e., establishing the liquid electrolytic solution over the solid medium cover, induced the vigorous development of gametophores well anchored in the solid medium [41]. The same authors reported spore dormancy and breaking dormancy to be unsuccessful when applying gibberellins conventionally as for breaking seed dormancy. However, again the imitation of environmental conditions, maintaining a temperature of over 25 °C for 3 months, produced over 90% germination rates for the spores treated that way. However, spore dormancy in bryophytes remains an obscure and unknown field of bryophyte biology.

In another rare and threatened species, also protected by the Bern Convention, namely *Pyramidula tetragona* (Brid.) Brid., spore dormancy seems to be related to the population the spores originated from. While some did have strong dormancy, others germinated rather well (Sabovljević et al., unpubl. results). 

Unexpectedly, some species need at higher temperatures for at least short periods to induce bud formation on the protonemal filaments. In high mountain rare moss species *Bartramia subulata* Bruch & Schimp., a three-day stimulus of over 25 °C induced numerous buds on the protonemal filaments and gametophore development (Sabovljevic et al., unpubl. results). Similarly, one-day exposure to a temperature of 0 °C, followed by a return to 18 °C, produced vigorous development of thali in liverwort *Marchantia polymorpha* L. subsp. *ruderalis* Bischl. & Boisselier (Sabovljevic et al., unpubl. results). It has also been documented that external factors affect the chemical constituents and contents of this species [42].

Externally applied chemicals can act differently on the species biology of bryophytes. Abscisic acid, for example, can provoke increased survival to various stresses [43,44]. The responses were dependent on the concentration applied, the time of application, the species and stress duration and stress intensity. 

Phenomena such as apogamy and apospory are rarely seen and documented in bryophytes. Apogamy is the development of sporophytes without the fusion of the gametes and without developing gametes at all, while apospory is the development of gametophytes (gametophores) from the vegetative cells derived from sporophytes without the intervention of spores. These phenomena are extremely rarely seen and reported [45]. Apogamous sporophyte formation in bryophytes was firstly reported in 1935 in *Phascum cuspidatum* Hedw., and the second record was rather recently [46] reported in *Fissidens crassipes* Wilson ex Bruch & Schimp. subsp. *warnstorfii* (M.Fleisch.) Brugg.-Nann. Both reports stated the absence of gametangia; however, there are no reports on spore development. There were two reports on the laboratory induction of apospory (in the moss *Amblystegium serpens* Schimp. [47] and *E. hungaricus* [29]). Sabovljevic et al. [29] stated that light condition is the main trigger for apogamy induction in *E. hungaricus* as previously speculated. In addition to light quality and intensity, other conditions can be included in apogamy development, such as hydration, sugars, chloral hydrate, growth regulators, inorganic nutrients, and endogenous factors, which may differ from species to species [38]. In *E. hungaricus,* the apogamous sporophyte produced spores which were able to germinate and develop new sporphytes directly {see the details in [29]}. The same authors inferred that low light intensity simulates one of the main resources of abduction (e.g., overgrowing by other plants), and thus acts as a trigger for rapid ontogenesic termination, i.e., speeding up the development by skipping up energetically expensive and time costly gametangia development. The apogamous sporophyte developed from green leafy gametophores or directly from spores in *E. hungaricus,* while that in *A. serpens* developed directly on primary protonemal filaments. 

Apospory in bryophytes is to some extent rather more reported than apogamy. However, induction, controls, signaling, the mechanisms of development and the ecological and biological significances of this phenomenon are rather obscured. The reproductive biology of bryophytes, even of well-known species, remains an unknown and poorly understood area [48] and one of the most significant in the conservation of bryophytes. Hence, the urgency of the need to increase research into the conservation physiology of hornworts, liverworts and mosses. 

Biotic agents in conservation physiology also remain less known in bryophytes compared to vascular plants. Interaction with some organisms such as lichens can lead to changes in the ploidy level in mosses [49], or even induce endoreduplication i.e., endopolyploidy [50], which can act as an evolutionary drive and lead to speciation or simply overcoming biotic stress and resisting the biotically caused effects. The experiments with *Bryum argenteum*, which developed very well on MS media type, showed a suppressive effect by introducing only one shoot of related *Bryum capillare* Hedw., suggesting interspecies communication, which is still unclear (Sabovljevic et al., unpubl. results).

Additionally, the report on endobionts in bryophytes (many of which are unknown species for science) shows that they seem to play a significant role in the development of some species (e.g., unknown fungi form the Helotiales group identified by DNA sequences present in *Sphagnum palustre* L.; [51]). Endobiotic fungi can pose a problem in establishing and maintaining in vitro cultures of many species, but the cohabitation of fungi and bryophytes seems to be very important. This can also be related to the establishment of vascular plants in nature and is of crucial significance for ecosystem restauration through the delivery of mycorhizal inoculum from liverworts to angiosperms [52].

The interspecific relationships among bryophytes also seem to be very important and such tests can be crucial. The field experiments with rare and threatened *Hamatocaulis vernicosus* (Mitt.) Hedenas, reintroduced into Hungary (Figure 1E), showed difficulties in survival in the presence of another moss species, namely *Calliergonella cuspidata* (Hedw.) Loeske, in sites where habitat quality is suboptimal, while in another site, it survived and spread rather well [53].

For the species growing in wetland environments, the reintroduction did not require any special anchoring methodology, apart from keeping the material in the net nest of inert material. Similarly, in most cases, soil species will spread secondary protonema over the substrate if the substrate and surrounding air remains humid. However, anchoring epiphytic and epilithic species requires testing and this can be a crucial task prior to removing bryophytes from captivity conditions. The rare and threatened dendrothelmatic (water-filled tree hole) mosses *Anacamptodon splachnoides* (Brid.) Brid. and *Zygodon forsterii* (Dicks.) Mitt. (Figure 1A,H) can be attached by a small amount of semi-liquid media since they are constantly close to the water level in the tree hole (Sabovljević et al., unpubl. data), which maintains relative high humidity. However, for rocky species, such as *Anomodon rostratus* (Hedw.) Schimp. (Figure 1B), egg white and yogurt seem to offer the solution for rock anchoring, combined with high air humidity. These are crucial factors for achieving rock anchoring (Sabovljević et al., unpubl. data). In habitat types of general high humidity, the translocation and anchoring of Bern Convention species *Dicranum viride* Lindb. (Figure 1D) to the tree trunk was successful because of the tight cover with the network [54]. 

Well-preserved samples of herbarium moss specimens can be good initial material for the establishment and in vitro propagation of rare and threatened species. Such an example is *M. hornschuchiana* revived after 25 years from the sample deposited in the BONN herbarium originating from Switzerland [27]. No data on the survival of the propagules and spores from the herbarium samples are available. 

The development of gametes and sporophytes in laboratory conditions is a rather difficult task, bearing in mind that almost half of the species are dioecious, and that it is questionable whether one is dealing with male or female start material/spores. Additionally, many threatened species are assumed to have lost sexual reproduction and are documented as reproducing exclusively vegetatively. Additionally, problems can arise in the different physiology of male and female organisms. In addition to apogamy, sporophyte production in in vitro conditions is also known in rare mosses *Physcomitrium sphaericum* (Hedw.) Brid. and *P. eurystomum* Sendtn. (Sabovljević et al., unpubl. results). Gametes can be induced by certain combinations of plant growth regulators. In *B. argenteum*, for example, in in vitro conditions, fructose induced maleness, while certain auxins provoked the expression of femaleness [55]. 

Additional tests of the effects of emerging pollutants in the environment on rare bryophytes are needed since these can also be one of the reasons for the disappearance or decline of populations. Many inferred opinions are easily taken as being scientifically valid, but sometimes, unexpected features may be found. The response to heavy metals, tolerance and resistance seems to be species- or even genotype-specific (e.g., [56,57,58,59,60]). However, sometimes, some rare and threatened species can be unexpectedly found in toxically loaded habitats (e.g., *Helodium blandowii* (F. Weber & D. Mohr) Warnst [61,62]), indicating obscure knowledge on the functional traits of rare and threatened species and raising the significance of the further development of the conservation physiology of bryophytes. 

## 3. Conclusions

Modern conservation demands more active approaches after generating red lists and red data books. It should move to the next level of conservation beyond population rebuilding and habitat restoration. Modern conservation science should be more about documenting responses to stressors, ensuring that those achieved quickly are included in conservation management programs.

Apart from some efforts to stop the decline and success in the conservation of other groups of organisms, a recent report said that the biodiversity loss rate has not slowed down [63]. This is the result of our efforts focusing on selected groups or targeted species, so human efforts in conservation should also be spread to other, non-flagged organisms.

The emerging field of bryophyte conservation physiology should offer and develop solutions for the survival of threatened biological entities rather than identify problems which are often present in other areas of conservation sciences. 

Mechanism elucidation and documentation of the problems cannot be the goal of conservation physiology *per se*, but need to be clearly in the service of survival. This means that the aim should be the self-sustainability of the target biological entity. Since the functional biodiversity of bryophytes is a necessary pre-requisite for successful conservation, the assessment of physiological diversity through varieties of function and tolerances among individuals, populations or species should always be taken into account in protection and conservation programs cross-referenced with a combination of data, such as genetic structure, developmental features or environmental influences, where possible. 

Understanding optimal environmental conditions for bryophyte development in ex situ conditions should lead to successful captivity regeneration, propagation, and breeding, as well as the development of in vitro tissue culture collections and spore bank protocols for targeted geo- and genotypes.

The markers developed in conservation investigation should be applied in medium to long-term monitoring programs of bryophytes, which overlap with the goals that predictive models should also include physiological parameters in practice.

## Data Availability

Not applicable.
